# Patient-Reported Receipt of Satisfactory Healthy Lifestyle Guidance and Associated Factors Among Outpatients in China: A Nationwide Cross-Sectional Survey

**DOI:** 10.3390/healthcare14162635

**Published:** 2026-08-20

**Authors:** Jiarun Mi, Yuanli Liu

**Affiliations:** School of Health Policy and Management, Chinese Academy of Medical Sciences and Peking Union Medical College, Beijing 100730, China

**Keywords:** outpatient care, healthy lifestyle guidance, lifestyle counseling, patient satisfaction, patient experience, tertiary hospitals, China, cross-sectional study

## Abstract

**Highlights:**

**What are the main findings?**

- Satisfactory healthy lifestyle guidance was common among outpatients in China’s tertiary public hospitals.
- Communication, respectful care, and satisfaction with lifestyle guidance were closely correlated dimensions of patients’ overall outpatient experience.

**What are the implications of the main findings?**

- Lifestyle guidance should be integrated into routine outpatient workflows across multiple lifestyle domains.
- Lifestyle guidance should be evaluated within the broader context of patients’ communication and interpersonal care experiences.

**Abstract:**

**Background**: Healthy lifestyle guidance is an important component of outpatient preventive care, yet evidence is limited on whether outpatients receive satisfactory guidance across multiple lifestyle domains. This study examined receipt of satisfactory healthy lifestyle guidance and its associated factors among outpatients in China. **Methods**: This national multicenter cross-sectional study used data from the outpatient component of the fifth National Third-Party Evaluation of the Action Plan for Further Improving Healthcare Services. A total of 31,599 outpatients from 143 tertiary public hospitals across all 31 provincial-level administrative regions of mainland China were included. The primary outcome was receipt of satisfactory healthy lifestyle guidance, defined as receiving at least one type of guidance and reporting satisfaction or high satisfaction. Mixed-effects logistic regression models with hospital-level random intercepts were used to identify associated factors. **Results**: Dietary guidance was the most frequently reported domain, followed by physical activity, weight management, and smoking cessation and alcohol reduction guidance. Overall, most outpatients were classified as having received satisfactory healthy lifestyle guidance. In the fully adjusted model, male sex and hypertension were associated with higher odds of satisfactory guidance, whereas a greater number of chronic conditions and unclear physician type were associated with lower odds. Satisfaction with history taking and disease communication and perceived respect and comfort from healthcare professionals were strongly associated with satisfactory healthy lifestyle guidance. However, these contemporaneously measured patient-reported indicators may reflect overlapping dimensions of patients’ overall outpatient care experience. Sensitivity analyses yielded broadly consistent findings. **Conclusions**: Satisfactory healthy lifestyle guidance was common among outpatients in China’s tertiary public hospitals, but guidance was unevenly distributed across lifestyle domains. Patients’ evaluations of satisfactory healthy lifestyle guidance were closely associated with their broader communication and interpersonal care experiences. However, because these measures were self-reported and assessed contemporaneously, the observed associations should not be interpreted causally.

## 1. Introduction

Chronic noncommunicable diseases remain a major global public health challenge and are closely associated with modifiable lifestyle-related risk factors, including unhealthy diet, physical inactivity, tobacco use, harmful alcohol consumption, insufficient sleep, and inadequate weight management [1,2,3,4,5]. Healthy lifestyle guidance delivered by healthcare professionals is therefore an important component of health promotion, disease prevention, and chronic disease management [6,7,8,9].

Outpatient care provides repeated opportunities to deliver lifestyle guidance to a large and diverse patient population [10,11]. Clinical encounters may create opportunities for healthcare professionals to identify behavioral risks and provide brief, individualized recommendations [10,12]. However, evidence from ambulatory and primary care settings suggests that lifestyle counseling is not consistently delivered, and patients may continue to have unmet needs for advice on diet, physical activity, weight management, and smoking cessation [13,14,15,16]. Previous studies have also tended to focus on individual lifestyle behaviors or on whether counseling was provided, with less attention to patients’ satisfaction with guidance across multiple lifestyle domains. In China, outpatient satisfaction and general patient experience in tertiary hospitals have been widely examined [17,18]. A previous study based on the same national healthcare service evaluation described the provision of healthy lifestyle guidance and examined factors associated with overall satisfaction among outpatients and inpatients [19]. However, that study did not separately examine receipt of any guidance and satisfaction among guidance recipients, incorporate patient-reported communication and humanistic care experiences, account for hospital-level clustering, or investigate factors associated with receipt across specific lifestyle guidance domains. Therefore, evidence regarding the reach and patient-perceived quality of healthy lifestyle guidance in outpatient care remains limited.

From a patient-centered care perspective, satisfaction with lifestyle guidance may depend not only on whether guidance is provided, but also on whether the advice is understandable, relevant to patients’ health concerns, responsive to their needs and preferences, and delivered in a respectful and supportive manner [20,21,22,23]. Clinician–patient communication and humanistic care may therefore represent important process factors shaping patients’ perceptions of the quality and acceptability of lifestyle guidance [20,22,24]. Accordingly, satisfactory lifestyle guidance can be viewed as a patient-reported outcome influenced by patient characteristics, clinical needs, the outpatient visit context, and the quality of interpersonal care during the clinical encounter [21,22]. Receipt of guidance and satisfaction with the guidance received capture related but distinct dimensions of outpatient preventive care. Receipt indicates whether lifestyle guidance reached the patient, but does not reflect how the patient evaluated its relevance, acceptability, or perceived quality. Conversely, satisfaction can only be assessed among patients who received guidance and therefore does not capture the reach of the service [20,21,22,23]. We therefore used receipt of satisfactory healthy lifestyle guidance as an integrated patient-reported outcome to identify patients who both received at least one type of guidance and evaluated the guidance positively. This composite outcome was not intended to replace separate analyses of receipt and satisfaction. Because the two components represent distinct stages of care and may have different associated factors, we also examined receipt of any guidance and satisfaction among guidance recipients separately.

Using data from a large nationwide outpatient survey in China, this study aimed to describe the types of healthy lifestyle guidance received by outpatients, assess receipt of and satisfaction with such guidance, and identify factors associated with receipt of satisfactory healthy lifestyle guidance. We additionally examined receipt of any guidance and satisfaction among guidance recipients separately and conducted domain-specific analyses of five major types of lifestyle guidance.

## 2. Methods

### 2.1. Study Design and Population

This study was a secondary analysis of the outpatient component of a national, multicenter, cross-sectional survey in China and aimed to investigate the receipt of satisfactory healthy lifestyle guidance and its associated factors. Data were derived from the fifth National Third-Party Evaluation of the Action Plan for Further Improving Healthcare Services. Data from the same national evaluation were previously used to describe the provision of healthy lifestyle guidance and examine overall satisfaction with such guidance among outpatients and inpatients [19]. The outpatient sample in the present study substantially overlapped with that included in the previous publication. However, the present study addressed distinct research questions by constructing an integrated outcome combining receipt and satisfaction, separately examining receipt of guidance and satisfaction among recipients, incorporating communication and humanistic care measures, accounting for hospital-level clustering, and conducting domain-specific analyses. This national evaluation was commissioned by the National Health Commission of China and the National Administration of Traditional Chinese Medicine, and was organized and implemented by the School of Health Policy and Management, Peking Union Medical College [25]. The survey was carried out in the first quarter of 2021 and included tertiary public hospitals from all 31 provincial-level administrative regions of mainland China. In this study, the term “nationwide” refers to the geographic coverage of the survey across all 31 provincial-level administrative regions of mainland China and does not imply that the participating hospitals or patients constituted a nationally representative probability sample.

The survey used a two-stage sampling approach. First, hospitals were selected to reflect both nationwide geographic distribution and the major categories of tertiary public hospitals in China. From each provincial-level administrative region, one general hospital, one traditional Chinese medicine hospital, and one maternal and child health hospital were randomly sampled, giving 93 local tertiary public hospitals. The survey also included 43 tertiary hospitals affiliated with or administered by the former national health administrative authority and seven traditional Chinese medicine hospitals directly administered by the National Administration of Traditional Chinese Medicine. In total, the hospital sample consisted of 143 tertiary public hospitals, including general hospitals, traditional Chinese medicine hospitals, maternal and child health hospitals, and other specialty hospitals.

Within participating hospitals, eligible outpatients were recruited through convenience sampling by trained third-party investigators in designated outpatient areas. No complete patient-level sampling frame or individual selection probabilities were available. The minimum sample size was determined according to the evaluation protocol. Assuming an outpatient satisfaction rate of 85%, with a significance level of 0.05 and an allowable error of 0.05, at least 196 outpatients were required from each hospital. To account for possible non-response and invalid questionnaires, each hospital was asked to obtain no fewer than 200 valid outpatient questionnaires. Eligible respondents were patients who had completed all or most procedures of their outpatient visit on the survey day. Patients visiting emergency departments, special outpatient clinics, or international medical departments were excluded. When patients were unable to answer the questionnaire by themselves, such as children or some older adults, their guardians or accompanying family members were allowed to provide responses.

The outpatient questionnaire was designed by the School of Health Policy and Management, Peking Union Medical College. The instrument was developed through literature review, expert consultation, stakeholder discussion, pilot testing, and multiple rounds of revision. Its content validity was evaluated by experts, and previous psychometric testing supported its reliability and validity. Exploratory factor analysis showed that the factor loading of each item was above 0.50, and Cronbach’s alpha coefficients for all dimensions were greater than 0.75 [26]. Before data collection, standardized training was provided to provincial expert teams and field investigators, covering the study protocol, item interpretation, operational definitions, and questionnaire administration procedures. During the survey, provincial expert teams supervised fieldwork, monitored implementation progress, and conducted quality control. Investigators were required to follow standardized procedures, maintain a neutral attitude during questionnaire administration, check questionnaires for completeness, and reduce potential information bias.

A total of 39,870 outpatients were approached, of whom 34,021 completed and returned the questionnaire, yielding a response rate of 85.3%. Among the returned questionnaires, 31,622 met the prespecified survey validity criteria, corresponding to a questionnaire validity rate of 92.9%. For the current analysis, 23 respondents with missing information on receipt of or satisfaction with healthy lifestyle guidance were further excluded, resulting in a final analytical sample of 31,599 outpatients (Supplementary Figure S1). The study was approved by the Ethics Committee of Peking Union Medical College (Approval No. CAMS&PUMC-IEC-2021-033).

### 2.2. Study Variables

The primary outcome was receipt of satisfactory healthy lifestyle guidance during the outpatient visit. This outcome was constructed using two questionnaire items. First, participants were asked which types of healthy lifestyle guidance they had received from healthcare professionals during the outpatient visit. Multiple responses were allowed, including dietary guidance, smoking cessation and alcohol reduction guidance, physical activity guidance, weight management guidance, psychological knowledge and emotion-regulation guidance, sleep guidance, oral hygiene guidance, social activity guidance, cognitive activity guidance, eye health guidance, other lifestyle guidance, and no relevant guidance. Participants who reported receiving any type of healthy lifestyle guidance were further asked to rate their satisfaction with the guidance provided by healthcare professionals on a five-point Likert scale, ranging from very dissatisfied to very satisfied.

For descriptive analyses, receipt of and satisfaction with healthy lifestyle guidance were classified into six mutually exclusive categories: no guidance, very dissatisfied, dissatisfied, neutral, satisfied, and very satisfied. For the main regression analyses, a binary outcome was defined. Participants were classified as having received satisfactory healthy lifestyle guidance if they reported receiving at least one type of healthy lifestyle guidance and rated the guidance as satisfied or very satisfied. Participants who reported no relevant guidance, or who reported receiving guidance but rated it as neutral, dissatisfied, or very dissatisfied, were classified as not having received satisfactory healthy lifestyle guidance. This binary composite outcome was constructed specifically for the present analysis from the two questionnaire items and was not a separately validated multi-item scale. The development and psychometric evaluation described above applied to the overall outpatient questionnaire rather than specifically to this composite outcome.

Covariates were selected from the outpatient questionnaire and hospital-level survey information based on prior literature and conceptual relevance. Patient sociodemographic characteristics included age group, sex, senior high school education or above, household registration status, and annual household income. Hospital characteristics included hospital category and geographic region. Hospital category was classified as general hospital, traditional Chinese medicine hospital, maternal and child health hospital, or other specialty hospital. Geographic region was categorized as eastern, central, or western China according to the location of the sampled hospital. Disease-related variables included the number of self-reported chronic conditions and specific chronic diseases. The number of chronic conditions was calculated according to participants’ self-reported history of physician-diagnosed chronic diseases. Chronic disease status was based on participants’ self-reported history of physician-diagnosed conditions and was not independently verified using medical records, laboratory results, or other clinical documentation. Specific chronic diseases included hypertension, diabetes, cardiocerebrovascular disease, cancer, and chronic respiratory disease. Cardiovascular and cerebrovascular disease was defined as having coronary heart disease or stroke, and chronic respiratory disease was defined as having chronic obstructive pulmonary disease or asthma. Outpatient visit characteristics included the type of physician visited and the total cost of the outpatient visit. The type of physician visited was categorized as renowned specialist, chief physician, associate chief physician, attending physician, resident physician, or unknown. The total cost of the outpatient visit was categorized as ≤150 yuan, 150–400 yuan, or >400 yuan. Patient experience variables included satisfaction with communication about medical history and perceived respect and comfort from healthcare professionals. These variables were dichotomized into satisfied versus not satisfied, with responses of satisfied or very satisfied classified as satisfied and all other responses classified as not satisfied.

### 2.3. Statistical Analysis

Descriptive analyses were conducted to summarize the types of healthy lifestyle guidance received by outpatients. Because participants could select more than one type of guidance, each type was reported as the number and percentage of the total analytical sample. Receipt of, and satisfaction with, healthy lifestyle guidance were further summarized using six mutually exclusive categories: no guidance, very dissatisfied, dissatisfied, neutral, satisfied, and very satisfied. Participant characteristics were described across these categories, with categorical variables presented as numbers and percentages. Because sampling probabilities were unavailable at both the hospital and patient levels, sampling or post-stratification weights were not applied. All descriptive proportions reported in this study are therefore unweighted estimates for the surveyed sample and should not be interpreted as nationally representative prevalence estimates.

Missingness was assessed for all variables included in the analyses. After excluding 23 respondents with missing information on receipt of or satisfaction with healthy lifestyle guidance, there were no missing values for the covariates included in the primary regression models. Therefore, no additional participants were excluded because of missing covariate data, no imputation was performed, and Models 1–5 were all fitted using the same analytical sample of 31,599 participants. The “unknown physician type” category comprised respondents who were unable to identify the professional title of the physician consulted and was retained as a separate response category rather than treated as missing data.

For regression analyses, the binary outcome of receipt of satisfactory healthy lifestyle guidance was used. Univariable mixed-effects logistic regression models were first fitted to estimate the crude association between each candidate factor and receipt of satisfactory healthy lifestyle guidance. Considering that outpatients were clustered within hospitals, a hospital-level random intercept was included in all univariable models.

Sequential multivariable mixed-effects logistic regression models were then constructed to examine factors associated with receipt of satisfactory healthy lifestyle guidance. Model 1 included patient sociodemographic characteristics, including age group, sex, senior high school education or above, household registration status, and annual household income. Model 2 additionally included hospital characteristics, including hospital category and geographic region. Model 3 further included disease-related variables, including the number of chronic conditions, hypertension, diabetes, cardiocerebrovascular disease, cancer, and chronic respiratory disease. Model 4 additionally included outpatient visit characteristics, including the type of physician visited and the total cost of the outpatient visit. Model 5 further included patient experience variables, including satisfaction with communication about medical history and perceived respect and comfort from healthcare professionals. All multivariable models included a hospital-level random intercept to account for within-hospital clustering. Associations were expressed as odds ratios (ORs) with 95% confidence intervals (CIs). Exploratory interaction analyses were conducted using the fully adjusted mixed-effects logistic regression model. We examined interactions between sex and the number of chronic conditions, and between age group and satisfaction, with history taking and disease communication. Likelihood ratio tests were used to compare models with and without the interaction terms. All interaction models included a hospital-level random intercept and were fitted using the same analytical sample of 31,599 participants. To distinguish between the provision of lifestyle guidance and patients’ evaluation of the guidance received, two complementary analyses were conducted. First, receipt of any healthy lifestyle guidance was defined as reporting at least one type of guidance, irrespective of satisfaction. Second, among respondents who reported receiving at least one type of guidance, satisfaction with guidance was defined as reporting satisfied or very satisfied, whereas neutral, dissatisfied, and very dissatisfied responses were classified as not satisfied. Fully adjusted mixed-effects logistic regression models with hospital-level random intercepts were fitted for both supplementary outcomes using the same covariates as the primary analysis. In addition, domain-specific analyses were conducted for dietary guidance, physical activity guidance, weight management guidance, sleep guidance, and smoking cessation and alcohol reduction guidance. Receipt of each guidance domain was treated as a separate binary outcome, with respondents who reported receiving the corresponding type of guidance classified as 1 and all other respondents classified as 0. For each domain, a fully adjusted mixed-effects logistic regression model with a hospital-level random intercept was fitted using the same covariates as Model 5 of the primary analysis.

Two sensitivity analyses were performed to assess the robustness of the main findings. First, participants who reported neutral satisfaction with healthy lifestyle guidance were excluded, and the fully adjusted mixed-effects logistic regression model was refitted. This analysis was conducted because neutral responses may represent an ambiguous category, reflecting neither clear satisfaction nor dissatisfaction. Excluding these participants allowed us to assess whether the main findings were robust to the classification of neutral responses in the binary outcome. Second, conventional logistic regression models without hospital-level random intercepts were fitted to evaluate whether the results were sensitive to the use of multilevel modeling for within-hospital clustering.

All analyses were conducted using R software (version 4.2.0). Statistical tests were two-sided, and *p* values < 0.05 were considered statistically significant.

## 3. Results

### 3.1. Descriptive Characteristics

A total of 31,599 outpatients were included in the final analysis. As shown in Table 1, dietary guidance was the most commonly reported type of healthy lifestyle guidance (22,515, 71.3%), followed by physical activity guidance (13,540, 42.8%), weight management guidance (10,030, 31.7%), and smoking cessation and alcohol reduction guidance (9505, 30.1%). In the surveyed sample, 2925 outpatients (9.3%) reported receiving no relevant healthy lifestyle guidance.

As shown in Table S1, 20,175 outpatients (63.8%) were very satisfied and 7401 (23.4%) were satisfied with the healthy lifestyle guidance received, whereas 1007 (3.2%) reported neutral satisfaction, 77 (0.2%) were dissatisfied, and 14 (0.0%) were very dissatisfied. Accordingly, 27,576 outpatients in the surveyed sample (87.3%) were classified as having received satisfactory healthy lifestyle guidance. Most participants were aged 18–35 years (44.3%) or 36–59 years (34.0%), female (66.4%), had senior high school education or above (79.2%), and had urban household registration (71.2%). General hospitals accounted for the largest proportion of participants (40.6%), and nearly half were from hospitals in eastern China (47.7%). Most participants reported positive communication and humanistic care experiences, including satisfaction with history taking and disease communication (95.5%) and perceived respect and comfort from healthcare professionals (95.1%).

### 3.2. Univariable Analyses

**Figure 1:**
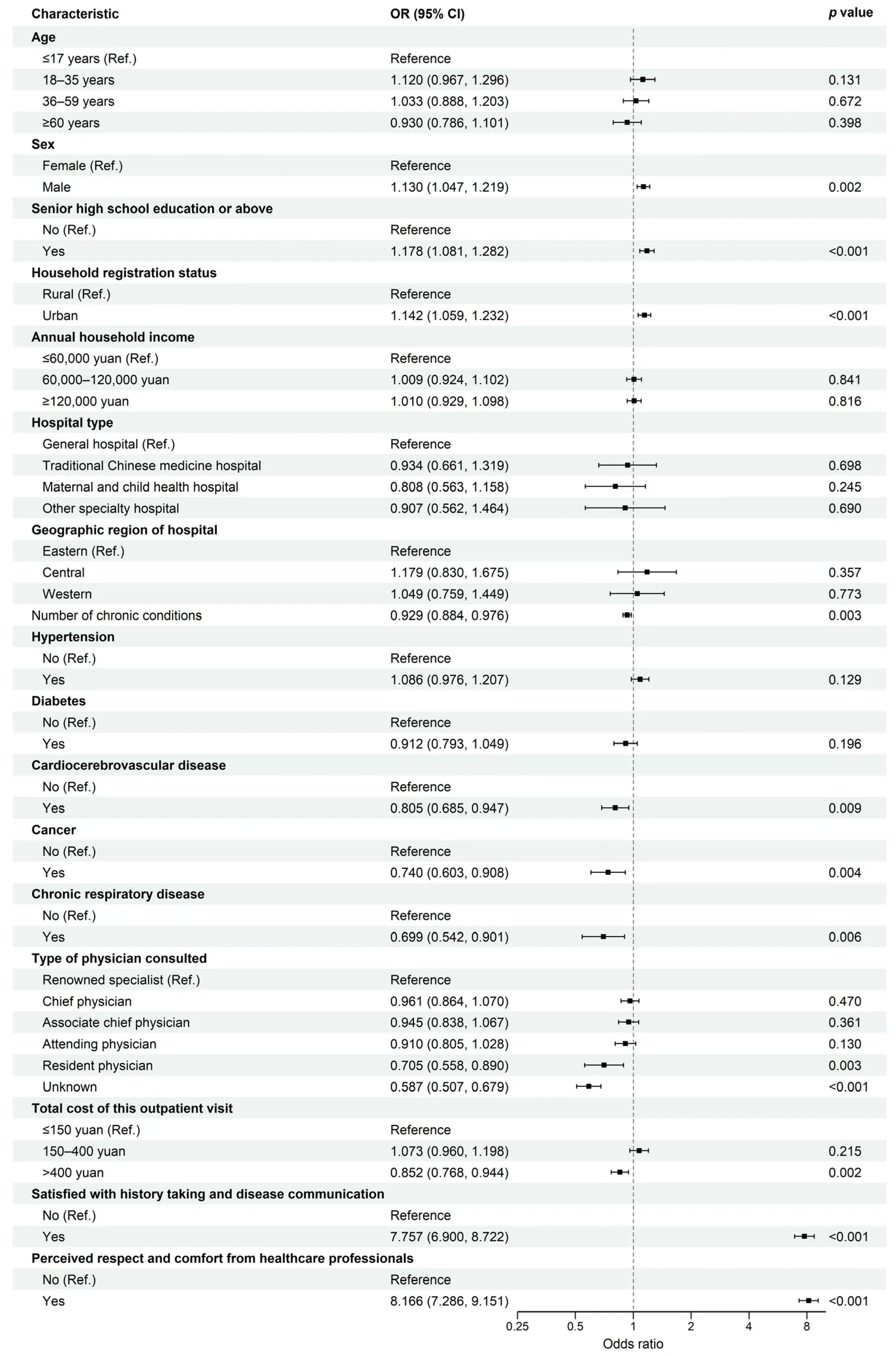
Univariable associations between patient characteristics and receipt of satisfactory healthy lifestyle guidance among outpatients. Black squares represent OR point estimates, horizontal lines represent 95% CIs, and the dashed vertical line indicates the null value (OR = 1).

As shown in Figure 1, univariable mixed-effects logistic regression models were fitted to estimate crude associations between candidate factors and receipt of satisfactory healthy lifestyle guidance. Male sex (OR = 1.130, 95% CI: 1.047–1.219, *p* = 0.002), senior high school education or above (OR = 1.178, 95% CI: 1.081–1.282, *p* < 0.001), and urban household registration (OR = 1.142, 95% CI: 1.059–1.232, *p* < 0.001) were associated with higher odds of receiving satisfactory healthy lifestyle guidance. In contrast, a greater number of chronic conditions (OR = 0.929, 95% CI: 0.884–0.976, *p* = 0.003), cardiocerebrovascular disease (OR = 0.805, 95% CI: 0.685–0.947, *p* = 0.009), cancer (OR = 0.740, 95% CI: 0.603–0.908, *p* = 0.004), chronic respiratory disease (OR = 0.699, 95% CI: 0.542–0.901, *p* = 0.006), visiting a resident physician (OR = 0.705, 95% CI: 0.558–0.890, *p* = 0.003), unclear physician type (OR = 0.587, 95% CI: 0.507–0.679, *p* < 0.001), and outpatient visit cost > 400 yuan (OR = 0.852, 95% CI: 0.768–0.944, *p* = 0.002) were associated with lower odds. The strongest crude associations were observed for satisfaction with history taking and disease communication (OR = 7.757, 95% CI: 6.900–8.722, *p* < 0.001) and perceived respect and comfort from healthcare professionals (OR = 8.166, 95% CI: 7.286–9.151, *p* < 0.001).

### 3.3. Multivariable Analyses

As shown in Table 2, sequential multivariable mixed-effects logistic regression models were constructed to examine factors associated with receipt of satisfactory healthy lifestyle guidance. Across the sequential models, male sex was consistently associated with higher odds of receiving satisfactory healthy lifestyle guidance. In the fully adjusted model, male outpatients had higher odds of receiving satisfactory guidance than female outpatients (OR = 1.206, 95% CI: 1.100–1.322, *p* < 0.001). Senior high school education or above showed a marginal positive association in the fully adjusted model (OR = 1.132, 95% CI: 0.998–1.285, *p* = 0.054). Urban household registration was associated with higher odds in Models 1–4, but the association was attenuated and no longer statistically significant after further adjustment for communication and humanistic care experience in Model 5 (OR = 1.092, 95% CI: 0.991–1.203, *p* = 0.075). Annual household income was not significantly associated with receipt of satisfactory healthy lifestyle guidance in the fully adjusted model. Hospital characteristics, including hospital type and geographic region, were not significantly associated with receipt of satisfactory healthy lifestyle guidance after multivariable adjustment. Regarding disease burden, a greater number of chronic conditions was associated with lower odds of receiving satisfactory guidance in Models 3–5. In the fully adjusted model, each additional chronic condition was associated with lower odds of receiving satisfactory guidance (OR = 0.853, 95% CI: 0.745–0.976, *p* = 0.021). In contrast, hypertension was associated with higher odds of receiving satisfactory guidance (OR = 1.448, 95% CI: 1.193–1.757, *p* < 0.001). Other specific chronic diseases, including diabetes, cardiocerebrovascular disease, cancer, and chronic respiratory disease, were not significantly associated with the outcome after full adjustment. For outpatient visit characteristics, compared with outpatients visiting renowned specialists, those who were unclear about the type of physician consulted had significantly lower odds of receiving satisfactory healthy lifestyle guidance in the fully adjusted model (OR = 0.626, 95% CI: 0.520–0.755, *p* < 0.001). Visiting resident physicians was associated with lower odds in Model 4, but this association was attenuated after further adjustment for communication and humanistic care experience in Model 5 (OR = 0.786, 95% CI: 0.593–1.043, *p* = 0.095). Total outpatient visit cost was not significantly associated with receipt of satisfactory guidance in the fully adjusted model. Communication and humanistic care experience showed the strongest associations with receipt of satisfactory healthy lifestyle guidance. After adjustment for patient sociodemographic characteristics, hospital characteristics, disease burden, and outpatient visit characteristics, satisfaction with history taking and disease communication was associated with substantially higher odds of receiving satisfactory guidance (OR = 4.052, 95% CI: 3.482–4.715, *p* < 0.001). Similarly, outpatients who perceived respect and comfort from healthcare professionals were more likely to receive satisfactory healthy lifestyle guidance (OR = 4.755, 95% CI: 4.108–5.505, *p* < 0.001). Exploratory interaction analyses showed no statistically significant interaction between sex and the number of chronic conditions (likelihood ratio χ^2^ = 0.041, df = 1, *p* for interaction = 0.839) or between age group and satisfaction with history taking and disease communication (likelihood ratio χ^2^ = 3.633, df = 3, *p* for interaction = 0.304). Detailed results are presented in Supplementary Table S2. In complementary analyses separating the two components of the primary outcome, the factors associated with receipt of any healthy lifestyle guidance and those associated with satisfaction among guidance recipients were partly different. Male sex, senior high school education or above, and hypertension were associated with higher odds of receiving any guidance, whereas annual household income of at least 120,000 yuan and unknown physician type were associated with lower odds. Among guidance recipients, residence in western China, a greater number of chronic conditions, consultation with a resident physician, and unknown physician type were associated with lower odds of satisfaction, whereas hypertension was associated with higher odds. Satisfaction with history taking and disease communication and perceived respect and comfort from healthcare professionals were associated with both outcomes, but the magnitudes of association were substantially greater for satisfaction among recipients than for receipt of any guidance. Detailed results are presented in Table 3. Domain-specific analyses further showed that the factors associated with receipt of lifestyle guidance varied across the five major guidance domains. Male sex was associated with higher odds of receiving smoking cessation and alcohol reduction guidance, but with lower odds of receiving weight management and sleep guidance. Disease-related factors also showed domain-specific patterns: hypertension, diabetes, cardiocerebrovascular disease, and cancer were associated with higher odds of receiving dietary guidance, whereas chronic respiratory disease was associated with higher odds of receiving smoking cessation and alcohol reduction guidance. Satisfaction with history taking and disease communication and perceived respect and comfort from healthcare professionals were positively associated with receipt across all five guidance domains. Detailed results are presented in Table S3.

### 3.4. Sensitivity Analyses

**Figure 2:**
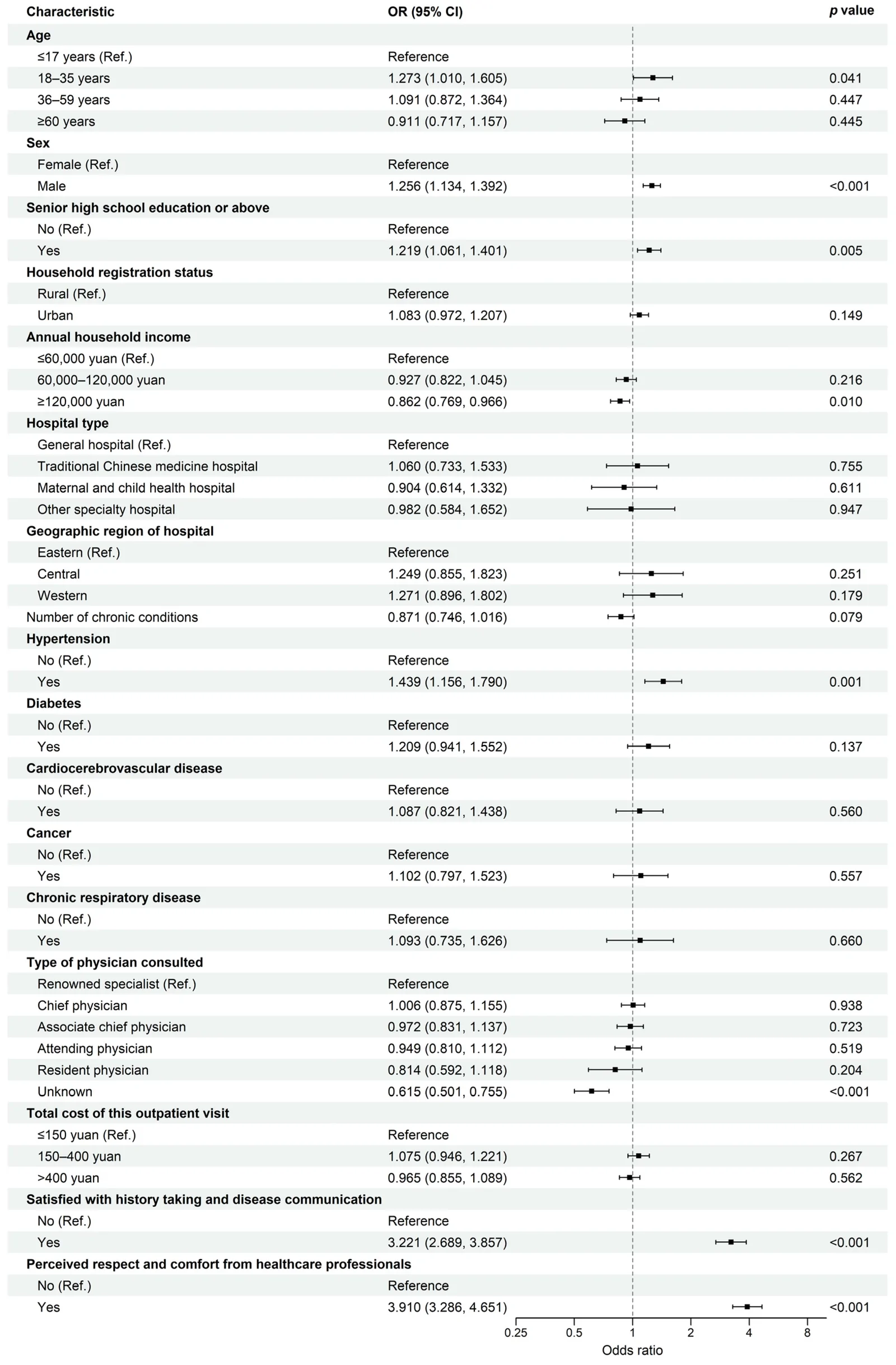
Sensitivity analysis of factors associated with receipt of satisfactory healthy lifestyle guidance after excluding outpatients with neutral responses. Black squares represent OR point estimates, horizontal lines represent 95% CIs, and the dashed vertical line indicates the null value (OR = 1).

As shown in Figure 2, after excluding outpatients with neutral satisfaction responses, the main findings remained generally consistent with the primary analysis. Male sex (OR = 1.256, 95% CI: 1.134–1.392, *p* < 0.001), senior high school education or above (OR = 1.219, 95% CI: 1.061–1.401, *p* = 0.005), and hypertension (OR = 1.439, 95% CI: 1.156–1.790, *p* = 0.001) were associated with higher odds of receiving satisfactory healthy lifestyle guidance. In contrast, annual household income ≥ 120,000 yuan (OR = 0.862, 95% CI: 0.769–0.966, *p* = 0.010) and unclear physician type (OR = 0.615, 95% CI: 0.501–0.755, *p* < 0.001) were associated with lower odds. Communication and humanistic care experience remained strongly associated with the outcome, including satisfaction with history taking and disease communication (OR = 3.221, 95% CI: 2.689–3.857, *p* < 0.001) and perceived respect and comfort from healthcare professionals (OR = 3.910, 95% CI: 3.286–4.651, *p* < 0.001).

**Figure 3:**
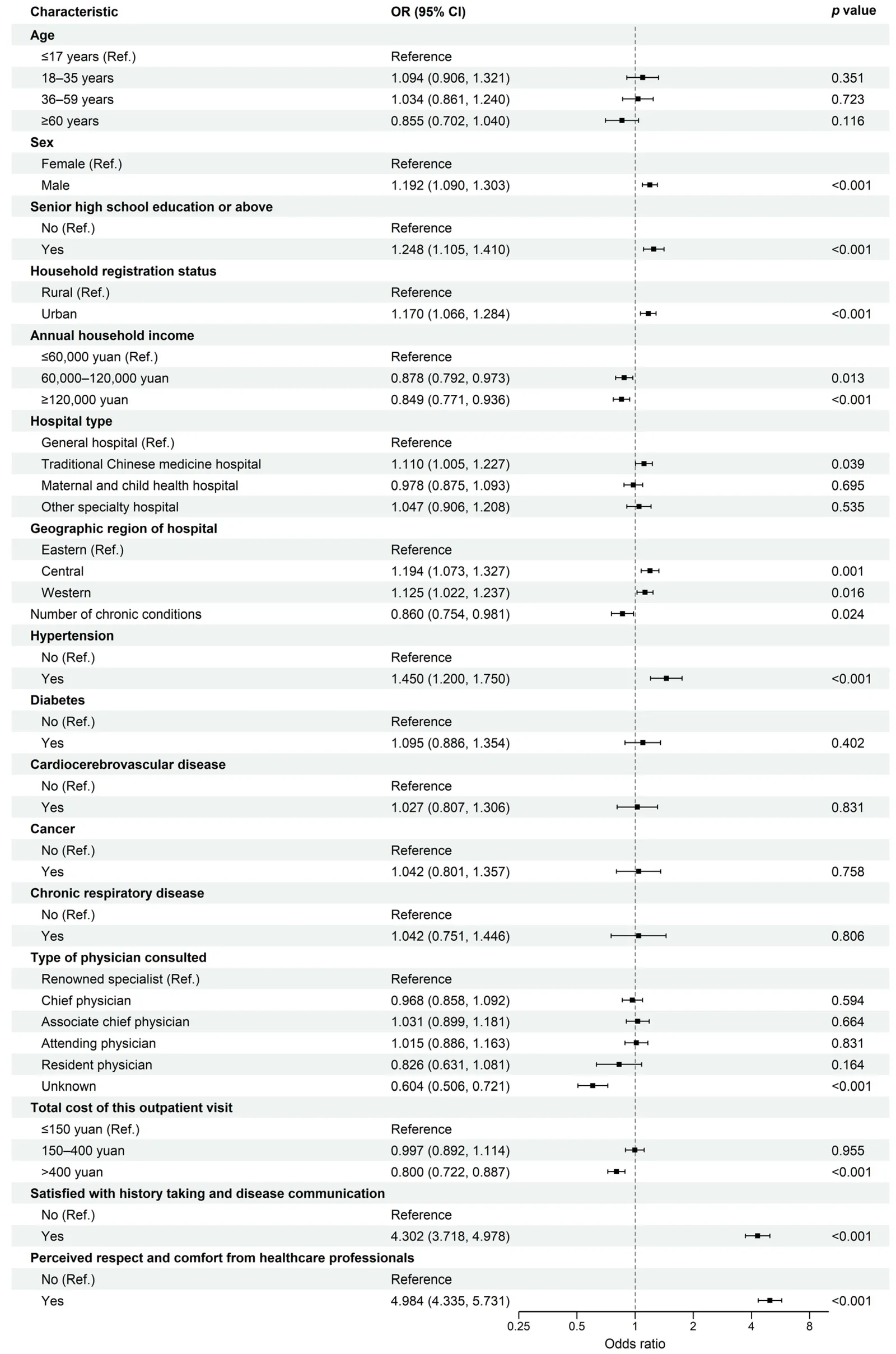
Sensitivity analysis of factors associated with receipt of satisfactory healthy lifestyle guidance using ordinary logistic regression. Black squares represent OR point estimates, horizontal lines represent 95% CIs, and the dashed vertical line indicates the null value (OR = 1).

As shown in Figure 3, ordinary logistic regression models without hospital-level random intercepts also yielded results broadly consistent with the main mixed-effects models. Male sex, senior high school education or above, urban household registration, and hypertension were associated with higher odds of receiving satisfactory healthy lifestyle guidance, whereas a greater number of chronic conditions, unclear physician type, and outpatient visit cost > 400 yuan were associated with lower odds. The strongest associations were again observed for satisfaction with history taking and disease communication (OR = 4.302, 95% CI: 3.718–4.978, *p* < 0.001) and perceived respect and comfort from healthcare professionals (OR = 4.984, 95% CI: 4.335–5.731, *p* < 0.001). Overall, these sensitivity analyses supported the robustness of the main findings.

## 4. Discussion

In this national multicenter cross-sectional study of 31,599 outpatients in China’s tertiary public hospitals, we found that most outpatients reported receiving healthy lifestyle guidance and were satisfied with the guidance received. Dietary guidance was the most frequently reported type of guidance, followed by physical activity guidance, weight management guidance, and smoking cessation and alcohol reduction guidance. Overall, 87.3% of outpatients were classified as having received satisfactory healthy lifestyle guidance. In multivariable analyses, male sex and hypertension were associated with higher odds of receiving satisfactory healthy lifestyle guidance, whereas a greater number of chronic conditions and being unclear about the type of physician consulted were associated with lower odds. Satisfaction with history taking and disease communication and perceived respect and comfort from healthcare professionals were strongly associated with receipt of satisfactory healthy lifestyle guidance. However, because these measures and the outcome were patient-reported and assessed contemporaneously, the observed relationships may reflect correlations among related dimensions of patients’ overall outpatient care experience. These findings were generally supported by sensitivity analyses, but they should not be interpreted as evidence that communication or humanistic care independently caused greater satisfaction with lifestyle guidance.

The distribution of healthy lifestyle guidance observed in this study suggests that outpatient lifestyle counseling in tertiary public hospitals in China is still mainly centered on traditional cardiometabolic risk factors. Dietary guidance was the most frequently reported type of guidance, followed by physical activity guidance, weight management guidance, and smoking cessation and alcohol reduction guidance. This pattern is broadly consistent with international recommendations that emphasize healthy diet, regular physical activity, tobacco avoidance, and weight control as core components of lifestyle-based prevention and chronic disease management [6,8,10]. However, previous studies from ambulatory and primary care settings in other countries have suggested that lifestyle counseling is often insufficiently delivered, with unmet patient needs for advice on diet, exercise, weight reduction, and smoking cessation [13,21]. Compared with these studies, the relatively high proportion of satisfactory guidance observed in our study may reflect the policy emphasis on health education and service improvement in China’s tertiary public hospitals, but it may also partly relate to differences in survey setting, patient population, and outcome definition. This relatively high proportion observed in the surveyed sample should be interpreted in the context of China’s healthcare system and the specific setting of this survey. The survey was conducted in tertiary public hospitals as part of the National Third-Party Evaluation of the Action Plan for Further Improving Healthcare Services, a national initiative that emphasized patient experience, health education, service quality, and continuous improvement in public hospitals. Tertiary public hospitals in China may have more standardized service processes, stronger administrative requirements, and greater capacity to provide health education than primary care institutions or lower-level hospitals. These characteristics may have contributed to the relatively high proportion of patients reporting satisfactory lifestyle guidance.

Nevertheless, this finding should be interpreted cautiously. The relatively high proportion observed in the surveyed sample may also partly reflect the broad definition of satisfactory guidance, which required receipt of at least one type of guidance combined with a rating of satisfied or very satisfied. In addition, the patient-reported nature of the outcome, the hospital-based survey setting, and possible social desirability or courtesy bias may have increased positive responses. Therefore, the findings should not be interpreted as indicating that lifestyle guidance was uniformly comprehensive, individualized, or sufficient across all lifestyle domains or outpatient settings. These contextual differences should also be considered when comparing the present findings with evidence from primary care settings or healthcare systems in other countries. Notably, guidance on psychological knowledge and emotion regulation, sleep, oral hygiene, social activity, cognitive activity, and eye health was much less commonly reported. This finding indicates that although lifestyle guidance was common, its content may remain uneven and weighted toward conventional chronic disease risk factors rather than a broader, multidimensional understanding of healthy lifestyles. In China, previous studies have mainly focused on outpatient satisfaction and general patient experience in tertiary hospitals [17,18,27]. A previous study based on the same national healthcare service evaluation described the provision of different types of healthy lifestyle guidance and examined overall satisfaction with such guidance among outpatients and inpatients [19]. The outpatient sample in the present study substantially overlapped with that included in the previous publication. Therefore, the descriptive findings regarding the frequency of different guidance domains should not be interpreted as evidence from an independent new sample. Rather, the present study extends the earlier work by constructing a composite outcome integrating receipt and satisfaction, separately examining receipt of any guidance and satisfaction among guidance recipients, incorporating patient-reported communication and humanistic care experiences, accounting for hospital-level clustering, and examining factors associated with receipt across specific lifestyle guidance domains.

The complementary analyses highlighted the importance of distinguishing between receipt of guidance and satisfaction with the guidance received. Receipt reflects whether lifestyle counseling was delivered, whereas satisfaction reflects patients’ subjective evaluation of the guidance. The associated factors differed partly between these two outcomes. Sociodemographic characteristics such as sex and education were more clearly associated with whether guidance was received, whereas geographic region, number of chronic conditions, and physician type were more clearly associated with satisfaction among recipients. Communication and humanistic care experience were associated with both outcomes, but their substantially larger associations with satisfaction among recipients suggest that these measures were particularly closely related to patients’ subjective evaluation of the outpatient encounter.

The domain-specific analyses further indicated that the associated factors were not uniform across different types of lifestyle guidance. In particular, demographic and disease-related characteristics showed different patterns across dietary, physical activity, weight management, sleep, and smoking- and alcohol-related guidance. These findings suggest that factors associated with the composite outcome should not be assumed to operate identically across all lifestyle guidance domains.

The strong associations observed for communication and humanistic care experience should be interpreted cautiously. Satisfaction with history taking and disease communication, perceived respect and comfort from healthcare professionals, and satisfaction with lifestyle guidance were all patient-reported measures collected from the same respondents at the same time using the same questionnaire. Therefore, these associations may partly reflect conceptual overlap, common-method variance, or a general tendency to evaluate multiple dimensions of the same outpatient encounter consistently [28]. They should be interpreted primarily as correlations among related dimensions of patients’ overall care experience rather than as evidence that communication or respectful care independently caused greater satisfaction with lifestyle guidance. The temporal direction of these relationships also cannot be established, because patients with a more favorable overall impression of the encounter may rate multiple aspects of care more positively. Nevertheless, the close relationship among these patient-reported dimensions is consistent with previous evidence showing that clinician–patient communication, empathy, respect, and supportive interactions are associated with patients’ perceptions of care quality and satisfaction [20,22,23,24,29]. Future longitudinal and intervention studies are needed to determine whether improvements in communication and interpersonal care lead to better delivery or evaluation of lifestyle guidance.

The associations between disease burden and receipt of satisfactory healthy lifestyle guidance also deserve attention. In our study, hypertension was associated with higher odds of receiving satisfactory guidance, whereas a greater number of chronic conditions was associated with lower odds. The positive association for hypertension is clinically plausible, because lifestyle modification is a cornerstone of hypertension prevention and management. International and Chinese hypertension guidelines consistently emphasize salt reduction, healthy dietary patterns, weight control, regular physical activity, alcohol restriction, and smoking cessation as essential non-pharmacological strategies for blood pressure control [30,31,32]. Therefore, patients with hypertension may be more likely to receive lifestyle advice during outpatient visits, particularly regarding diet, exercise, weight management, and smoking or alcohol use. By contrast, the inverse association with the number of chronic conditions may indicate that patients with multimorbidity have more complex and less adequately addressed needs. One possible explanation is that multimorbidity is often accompanied by competing clinical priorities, polypharmacy, fragmented care, and increased treatment burden, which may reduce the time or opportunity available for individualized lifestyle counseling during short outpatient encounters [33,34]. Patients with multiple chronic conditions may also require more tailored, coordinated, and feasible recommendations rather than generic lifestyle advice. However, the mechanisms underlying the observed association cannot be determined from the present cross-sectional data. These findings highlight the need to strengthen integrated lifestyle guidance for patients with multimorbidity, with attention to patients’ priorities, treatment burden, and ability to implement behavior change in daily life.

The associations of sociodemographic and outpatient visit characteristics with satisfactory healthy lifestyle guidance were more modest but still informative. Male outpatients had consistently higher odds of receiving satisfactory guidance. This finding may partly reflect the clinical salience of several lifestyle-related risk factors among men, such as smoking, alcohol use, overweight or obesity, and cardiovascular risk, which may prompt healthcare professionals to provide more lifestyle-related advice during outpatient encounters [35,36]. Education and urban household registration showed positive associations in earlier models, but these associations were attenuated after further adjustment for communication and humanistic care experience. This pattern suggests that socioeconomic differences in satisfactory lifestyle guidance may be partly mediated or explained by differences in patients’ ability to communicate health needs, understand medical advice, navigate hospital services, or perceive the quality of care received. Previous studies have similarly suggested that patient sociodemographic characteristics may shape outpatient experience and satisfaction, but these effects are often intertwined with communication quality, perceived respect, and service processes [37,38]. In addition, outpatients who were unclear about the type of physician consulted had lower odds of receiving satisfactory healthy lifestyle guidance. Although this variable should not be interpreted as a direct measure of physician competence, it may reflect weaker patient engagement, less clear communication, or a less established patient–physician relationship during the visit. Evidence on continuity of care and patient–physician relationships indicates that ongoing, identifiable, and communicative relationships with clinicians are associated with higher patient satisfaction and better perceived care [39,40]. Therefore, improving the clarity of physician information, strengthening explanation during consultations, and enhancing patient engagement may help improve patients’ perception and acceptance of lifestyle guidance in outpatient settings.

These findings have several implications for improving outpatient preventive care. The following practical considerations are informed by the existing literature and implementation frameworks and should be regarded as potential strategies for future evaluation rather than interventions directly tested in the present study. First, healthy lifestyle guidance should be embedded into routine outpatient workflows rather than delivered only as brief, opportunistic advice. Structured counseling frameworks, such as the 5A approach of assessing risk behaviors, advising change, agreeing on goals, assisting behavior change, and arranging follow-up, may help clinicians provide concise and patient-centered guidance within limited consultation time [41]. Second, because lifestyle guidance covers multiple domains, including diet, physical activity, smoking, alcohol use, weight management, sleep, psychological well-being, and other health-related behaviors, hospitals may consider developing standardized but flexible counseling tools tailored to common outpatient conditions and risk profiles. Third, lifestyle guidance should not rely solely on physicians. Multidisciplinary support from nurses, health managers, dietitians, pharmacists, rehabilitation professionals, and mental health professionals may help translate medical advice into feasible self-management plans, especially for patients with multimorbidity or complex needs. This is consistent with chronic care and integrated NCD management models, which emphasize self-management support, team-based care, decision support, and linkage with community resources [42]. Fourth, digital tools, pre-consultation questionnaires, patient education materials, and post-visit follow-up may help identify lifestyle risks, provide standardized health education, and reduce the burden on clinicians, but they should complement rather than replace respectful and individualized clinician–patient communication. Finally, implementation should be evaluated not only by whether guidance is provided, but also by its reach, quality, patient acceptability, feasibility, and sustainability in real-world outpatient settings [43].

This study has several strengths. First, it was based on a large-scale national survey covering tertiary public hospitals across all 31 provincial-level administrative regions of mainland China, providing a broad overview of healthy lifestyle guidance in routine outpatient care. Second, the present study extends previous work based on the same national evaluation by distinguishing receipt of guidance from satisfaction among recipients, incorporating patient-reported communication and humanistic care measures, and conducting domain-specific multivariable analyses. Third, multiple domains of lifestyle guidance were assessed, including diet, physical activity, weight management, smoking and alcohol use, sleep, psychological regulation, oral hygiene, social activity, cognitive activity, and eye health. Fourth, mixed-effects logistic regression models were used to account for the clustering of outpatients within hospitals, and sensitivity analyses supported the robustness of the main findings.

Several limitations should also be noted. First, the cross-sectional design precludes causal inference, and we cannot determine whether better communication and humanistic care led to more satisfactory lifestyle guidance or whether patients with more positive overall experiences were more likely to report satisfaction with guidance. Second, receipt of guidance, satisfaction with guidance, chronic disease history, and patient experience were self-reported, which may have introduced recall bias, reporting bias, social desirability bias, or misclassification of disease status. Chronic disease history was not independently verified using medical records, laboratory results, or other clinical documentation. Third, the survey was conducted during the first quarter of 2021, when COVID-19-related infection-control measures and changes in outpatient service delivery may have affected outpatient attendance, patient composition, clinical workflows, clinician–patient communication, and the delivery of healthy lifestyle guidance. Therefore, the findings may not fully reflect routine outpatient practice during non-pandemic periods. Fourth, although the survey covered all 31 provincial-level administrative regions of mainland China, it did not use a nationally representative probability sample of outpatients. Hospital selection combined random and purposive components, and patients within participating hospitals were recruited through convenience sampling without a complete patient-level sampling frame. Although the response rate was relatively high at 85.3%, potential non-response and selection bias cannot be excluded. Patients who declined participation, left the hospital before being approached, or were too unwell or otherwise unable to complete the questionnaire may have differed systematically from respondents in terms of health status, care experience, or satisfaction. Because individual-level information on non-respondents was unavailable, respondents and non-respondents could not be compared, and the direction or magnitude of any resulting bias could not be assessed. In addition, because sampling probabilities were unavailable, sampling or post-stratification weights could not be applied. Consequently, the reported proportions are unweighted estimates for the surveyed sample and should not be interpreted as nationally representative prevalence estimates. Fifth, the study included only tertiary public hospitals; therefore, the findings may not be directly generalizable to primary care institutions, private hospitals, rural or lower-level healthcare facilities, or healthcare systems with different organizational and service-delivery characteristics.

## 5. Conclusions

In this nationwide cross-sectional study of outpatients in China’s tertiary public hospitals, receipt of satisfactory healthy lifestyle guidance was common, with dietary, physical activity, weight management, and smoking- and alcohol-related guidance being the most frequently reported domains. However, the distribution of guidance across lifestyle domains was uneven, suggesting that broader aspects of healthy lifestyles may still be insufficiently addressed in routine outpatient care. Receipt of satisfactory guidance was associated with patient characteristics, disease burden, and outpatient visit factors. Patients’ evaluations of lifestyle guidance were also strongly correlated with their broader communication and interpersonal care experiences. Because these measures were self-reported and assessed contemporaneously, these relationships should be interpreted as correlations among related dimensions of perceived care quality rather than as independent or causal effects. Future longitudinal and intervention studies are needed to clarify whether and how communication and interpersonal care influence the delivery and evaluation of lifestyle guidance.

## Figures and Tables

**Table 1:** Types of lifestyle guidance received among outpatients.

Type of Lifestyle Guidance	Outpatients, *n* (%)
Dietary guidance	22,515 (71.3)
Smoking cessation and alcohol reduction guidance	9505 (30.1)
Physical activity guidance	13,540 (42.8)
Weight management guidance	10,030 (31.7)
Psychological knowledge and emotion regulation guidance	7572 (24.0)
Sleep guidance	6759 (21.4)
Oral hygiene guidance	3009 (9.5)
Social activity guidance	1178 (3.7)
Cognitive activity guidance	963 (3.0)
Eye health guidance	2002 (6.3)
Other lifestyle guidance	145 (0.5)
No relevant guidance	2925 (9.3)

**Table 2:** Sequential multivariable mixed-effects logistic regression analyses of factors associated with receipt of satisfactory lifestyle guidance.

Category	Characteristic	Model 1 OR (95% CI)	Model 1 p Value	Model 2 OR (95% CI)	Model 2 p Value	Model 3 OR (95% CI)	Model 3 p Value	Model 4 OR (95% CI)	Model 4 p Value	Model 5 OR (95% CI)	Model 5 p Value
Patient sociodemographic characteristics	Age										
	≤17 years (Ref.)	Reference		Reference		Reference		Reference		Reference	
	18–35 years	1.092 (0.916, 1.303)	0.327	1.088 (0.912, 1.297)	0.351	1.087 (0.911, 1.297)	0.352	1.211 (0.991, 1.480)	0.061	1.184 (0.961, 1.459)	0.113
	36–59 years	1.003 (0.847, 1.189)	0.968	0.997 (0.840, 1.182)	0.969	0.999 (0.841, 1.186)	0.987	1.050 (0.865, 1.275)	0.621	1.037 (0.847, 1.270)	0.727
	≥60 years	0.900 (0.754, 1.074)	0.242	0.894 (0.748, 1.067)	0.214	0.898 (0.746, 1.081)	0.254	0.934 (0.758, 1.152)	0.524	0.917 (0.738, 1.141)	0.439
	Sex										
	Female (Ref.)	Reference		Reference		Reference		Reference		Reference	
	Male	1.161 (1.074, 1.254)	<0.001	1.158 (1.072, 1.251)	<0.001	1.158 (1.071, 1.251)	<0.001	1.189 (1.089, 1.299)	<0.001	1.206 (1.100, 1.322)	<0.001
	Senior high school education or above										
	No (Ref.)	Reference		Reference		Reference		Reference		Reference	
	Yes	1.097 (0.985, 1.221)	0.092	1.097 (0.985, 1.222)	0.092	1.100 (0.988, 1.226)	0.082	1.066 (0.945, 1.204)	0.300	1.132 (0.998, 1.285)	0.054
	Household registration status										
	Rural (Ref.)	Reference		Reference		Reference		Reference		Reference	
	Urban	1.154 (1.064, 1.251)	<0.001	1.153 (1.063, 1.251)	<0.001	1.152 (1.062, 1.249)	<0.001	1.124 (1.025, 1.233)	0.013	1.092 (0.991, 1.203)	0.075
	Annual household income										
	≤60,000 yuan (Ref.)	Reference		Reference		Reference		Reference		Reference	
	60,000–120,000 yuan	0.966 (0.883, 1.057)	0.456	0.967 (0.884, 1.058)	0.462	0.963 (0.881, 1.054)	0.417	0.964 (0.871, 1.068)	0.487	0.928 (0.834, 1.033)	0.171
	≥120,000 yuan	0.947 (0.868, 1.034)	0.223	0.949 (0.870, 1.035)	0.236	0.948 (0.869, 1.035)	0.234	0.943 (0.854, 1.040)	0.239	0.914 (0.824, 1.013)	0.085
Hospital characteristics	Hospital type										
	General hospital (Ref.)	—	—	Reference		Reference		Reference		Reference	
	Traditional Chinese medicine hospital	—	—	0.949 (0.672, 1.339)	0.765	0.953 (0.675, 1.344)	0.782	0.971 (0.683, 1.378)	0.868	1.004 (0.721, 1.396)	0.983
	Maternal and child health hospital	—	—	0.813 (0.567, 1.165)	0.260	0.811 (0.566, 1.162)	0.253	0.778 (0.538, 1.124)	0.181	0.852 (0.602, 1.205)	0.364
	Other specialty hospital	—	—	0.934 (0.575, 1.516)	0.782	0.942 (0.580, 1.530)	0.811	1.023 (0.622, 1.681)	0.929	1.028 (0.644, 1.641)	0.909
	Geographic region of hospital										
	Eastern (Ref.)	—	—	Reference		Reference		Reference		Reference	
	Central	—	—	1.156 (0.812, 1.647)	0.421	1.160 (0.814, 1.652)	0.412	1.178 (0.821, 1.690)	0.375	1.200 (0.854, 1.686)	0.294
	Western	—	—	1.057 (0.764, 1.463)	0.737	1.055 (0.763, 1.460)	0.745	1.023 (0.735, 1.426)	0.892	1.074 (0.786, 1.468)	0.653
Disease burden	Number of chronic conditions	—	—	—	—	0.853 (0.760, 0.957)	0.007	0.829 (0.729, 0.941)	0.004	0.853 (0.745, 0.976)	0.021
	Hypertension										
	No (Ref.)	—	—	—	—	Reference		Reference		Reference	
	Yes	—	—	—	—	1.417 (1.200, 1.674)	<0.001	1.484 (1.234, 1.785)	<0.001	1.448 (1.193, 1.757)	<0.001
	Diabetes										
	No (Ref.)	—	—	—	—	Reference		Reference		Reference	
	Yes	—	—	—	—	1.082 (0.898, 1.305)	0.407	1.066 (0.867, 1.310)	0.547	1.067 (0.859, 1.327)	0.556
	Cardiocerebrovascular disease										
	No (Ref.)	—	—	—	—	Reference		Reference		Reference	
	Yes	—	—	—	—	0.965 (0.779, 1.194)	0.741	1.036 (0.818, 1.311)	0.770	1.042 (0.813, 1.335)	0.744
	Cancer										
	No (Ref.)	—	—	—	—	Reference		Reference		Reference	
	Yes	—	—	—	—	0.912 (0.719, 1.157)	0.449	1.006 (0.770, 1.315)	0.964	0.970 (0.732, 1.285)	0.831
	Chronic respiratory disease										
	No (Ref.)	—	—	—	—	Reference		Reference		Reference	
	Yes	—	—	—	—	0.862 (0.647, 1.150)	0.313	0.860 (0.629, 1.177)	0.346	0.972 (0.694, 1.361)	0.870
Outpatient visit characteristics	Type of physician consulted										
	Renowned specialist (Ref.)	—	—	—	—	—	—	Reference		Reference	
	Chief physician	—	—	—	—	—	—	0.927 (0.822, 1.047)	0.222	0.955 (0.842, 1.083)	0.473
	Associate chief physician	—	—	—	—	—	—	0.918 (0.801, 1.053)	0.222	0.964 (0.836, 1.112)	0.618
	Attending physician	—	—	—	—	—	—	0.871 (0.758, 1.000)	0.050	0.903 (0.782, 1.043)	0.166
	Resident physician	—	—	—	—	—	—	0.675 (0.519, 0.879)	0.003	0.786 (0.593, 1.043)	0.095
	Unknown	—	—	—	—	—	—	0.569 (0.477, 0.679)	<0.001	0.626 (0.520, 0.755)	<0.001
	Total cost of this outpatient visit										
	≤150 yuan (Ref.)	—	—	—	—	—	—	Reference		Reference	
	150–400 yuan	—	—	—	—	—	—	1.070 (0.958, 1.196)	0.231	1.062 (0.946, 1.192)	0.306
	>400 yuan	—	—	—	—	—	—	0.853 (0.768, 0.947)	0.003	0.921 (0.826, 1.027)	0.139
Communication and humanistic care experience	Satisfied with history taking and disease communication										
	No (Ref.)	—	—	—	—	—	—	—	—	Reference	
	Yes	—	—	—	—	—	—	—	—	4.052 (3.482, 4.715)	<0.001
	Perceived respect and comfort from healthcare professionals										
	No (Ref.)	—	—	—	—	—	—	—	—	Reference	
	Yes	—	—	—	—	—	—	—	—	4.755 (4.108, 5.505)	<0.001

Notes: Model 1 included patient sociodemographic characteristics. Model 2 additionally adjusted for hospital characteristics. Model 3 further adjusted for disease burden. Model 4 additionally adjusted for outpatient visit characteristics. Model 5 further adjusted for communication and humanistic care experience. All models included 31,599 participants, and no observations were excluded because of missing covariate data.

**Table 3:** Fully adjusted mixed-effects logistic regression analyses of factors associated with receipt of any healthy lifestyle guidance and satisfaction among guidance recipients.

Category	Characteristic	Level	Receipt of Any Healthy Lifestyle Guidance		Satisfaction Among Guidance Recipients	
			OR (95% CI)	p Value	OR (95% CI)	p Value
Patient sociodemographic characteristics	Age	≤17 years	Reference		Reference	
		18–35 years	1.226 (0.974, 1.543)	0.083	1.120 (0.767, 1.634)	0.558
		36–59 years	1.055 (0.845, 1.317)	0.639	1.077 (0.743, 1.562)	0.694
		≥60 years	0.871 (0.687, 1.105)	0.256	1.132 (0.754, 1.700)	0.549
	Sex	Female	Reference		Reference	
		Male	1.276 (1.151, 1.414)	<0.001	1.017 (0.860, 1.202)	0.847
	Senior high school education or above	No	Reference		Reference	
		Yes	1.265 (1.102, 1.453)	<0.001	0.843 (0.662, 1.074)	0.167
	Household registration status	Rural	Reference		Reference	
		Urban	1.049 (0.941, 1.170)	0.386	1.144 (0.963, 1.361)	0.127
	Annual household income	≤60,000 yuan	Reference		Reference	
		60,000–120,000 yuan	0.921 (0.817, 1.039)	0.181	0.916 (0.756, 1.110)	0.370
		≥120,000 yuan	0.843 (0.752, 0.944)	0.003	1.088 (0.895, 1.322)	0.400
Hospital characteristics	Hospital type	General hospital	Reference		Reference	
		Traditional Chinese medicine hospital	1.036 (0.713, 1.507)	0.851	0.988 (0.715, 1.366)	0.941
		Maternal and child health hospital	0.884 (0.597, 1.309)	0.539	0.761 (0.539, 1.074)	0.120
		Other specialty hospital	0.941 (0.556, 1.593)	0.821	1.176 (0.728, 1.898)	0.508
	Geographic region of hospital	Eastern	Reference		Reference	
		Central	1.318 (0.898, 1.935)	0.159	0.916 (0.652, 1.286)	0.612
		Western	1.317 (0.924, 1.876)	0.127	0.711 (0.524, 0.964)	0.028
Disease burden	Number of chronic conditions	Per one-condition increase	0.948 (0.811, 1.107)	0.498	0.693 (0.555, 0.865)	0.001
	Hypertension	No	Reference		Reference	
		Yes	1.317 (1.057, 1.640)	0.014	1.597 (1.140, 2.237)	0.007
	Diabetes	No	Reference		Reference	
		Yes	1.155 (0.897, 1.486)	0.263	0.931 (0.648, 1.339)	0.701
	Cardiocerebrovascular disease	No	Reference		Reference	
		Yes	1.096 (0.824, 1.457)	0.529	1.125 (0.735, 1.723)	0.587
	Cancer	No	Reference		Reference	
		Yes	1.165 (0.839, 1.616)	0.362	0.726 (0.457, 1.154)	0.176
	Chronic respiratory disease	No	Reference		Reference	
		Yes	1.235 (0.815, 1.872)	0.319	0.843 (0.504, 1.408)	0.513
Outpatient visit characteristics	Type of physician consulted	Renowned specialist	Reference		Reference	
		Chief physician	0.997 (0.868, 1.146)	0.972	0.812 (0.637, 1.036)	0.094
		Associate chief physician	0.984 (0.841, 1.151)	0.841	0.937 (0.714, 1.230)	0.640
		Attending physician	0.957 (0.817, 1.122)	0.588	0.819 (0.623, 1.076)	0.152
		Resident physician	0.913 (0.662, 1.259)	0.579	0.612 (0.380, 0.985)	0.043
		Unknown	0.627 (0.511, 0.769)	<0.001	0.627 (0.444, 0.887)	0.008
	Total cost of this outpatient visit	≤150 yuan	Reference		Reference	
		150–400 yuan	1.060 (0.933, 1.206)	0.370	1.025 (0.825, 1.274)	0.822
		>400 yuan	0.943 (0.835, 1.065)	0.346	0.834 (0.683, 1.020)	0.077
Communication and humanistic care experience	Satisfied with history taking and disease communication	No	Reference		Reference	
		Yes	2.245 (1.881, 2.680)	<0.001	6.239 (5.063, 7.688)	<0.001
	Perceived respect and comfort from healthcare professionals	No	Reference		Reference	
		Yes	2.640 (2.225, 3.133)	<0.001	7.240 (5.913, 8.866)	<0.001

## Data Availability

The data presented in this study are available from the corresponding author upon reasonable request. The data are not publicly available due to privacy and ethical restrictions.

## Supplementary Materials

The following supporting information can be downloaded at: https://www.mdpi.com/article/10.3390/healthcare14162635/s1, Figure S1: Flow diagram of outpatient recruitment and derivation of the final analytical sample; Table S1: Characteristics of outpatients according to receipt of and satisfaction with healthy lifestyle guidance; Table S2: Exploratory interaction analyses for receipt of satisfactory healthy lifestyle guidance; Table S3: Fully adjusted mixed-effects logistic regression analyses of factors associated with receipt of specific healthy lifestyle guidance domains.
