# Supplementary material for: Patient-Reported Receipt of Satisfactory Healthy Lifestyle Guidance and Associated Factors Among Outpatients in China: A Nationwide Cross-Sectional Survey
Source: Healthcare (Basel). 2026 Aug 20;14(16):2635. doi: 10.3390/healthcare14162635 (PMC13511949; doi:10.3390/healthcare14162635)
Supplement: Supplementary file 1 [file healthcare-14-02635-s001.zip › healthcare-4418349-supplementary.pdf]

Patient-Reported Receipt of Satisfactory Healthy Lifestyle Guidance and Associated Factors Among Outpatients in China: A Nationwide Cross-Sectional Survey

Jiarun Mi [1] and Yuanli Liu [1*]

[1] School of Health Policy and Management, Chinese Academy of Medical Sciences and Peking Union Medical College, Dongdansantiao, Dongcheng district, Beijing 100730, China

* Correspondence: liuyuanli_pumc@163.com

Supplementary materials

Table S1. Characteristics of outpatients according to receipt of and satisfaction with healthy lifestyle guidance

Category	Characteristic	No guidance (N=2,925, 9.3%)	Very dissatisfied (N=14, 0.0%)	Dissatisfied (N=77, 0.2%)	Neutral (N=1,007, 3.2%)	Satisfied (N=7,401, 23.4%)	Very satisfied (N=20,175, 63.8%)	Overall (N=31,599, 100.0%)
Patient sociodemographic characteristics	**Age**							
	≤17 years	265 (10.4)	0 (0.0)	6 (0.2)	89 (3.5)	646 (25.3)	1,543 (60.5)	2,549 (8.1)
	18–35 years	1,224 (8.7)	6 (0.0)	38 (0.3)	456 (3.3)	3,332 (23.8)	8,933 (63.9)	13,989 (44.3)
	36–59 years	955 (8.9)	4 (0.0)	23 (0.2)	341 (3.2)	2,467 (23.0)	6,944 (64.7)	10,734 (34.0)
	≥60 years	481 (11.1)	4 (0.1)	10 (0.2)	121 (2.8)	956 (22.1)	2,755 (63.7)	4,327 (13.7)
	**Sex**							
	Female	2,036 (9.7)	7 (0.0)	42 (0.2)	660 (3.1)	4,834 (23.0)	13,402 (63.9)	20,981 (66.4)
	Male	889 (8.4)	7 (0.1)	35 (0.3)	347 (3.3)	2,567 (24.2)	6,773 (63.8)	10,618 (33.6)
	**Senior high school education or above**							
	No	770 (11.7)	5 (0.1)	9 (0.1)	215 (3.3)	1,599 (24.3)	3,990 (60.6)	6,588 (20.8)
	Yes	2,155 (8.6)	9 (0.0)	68 (0.3)	792 (3.2)	5,802 (23.2)	16,185 (64.7)	25,011 (79.2)
	**Household registration status**							
	Rural	953 (10.5)	5 (0.1)	34 (0.4)	354 (3.9)	2,330 (25.6)	5,421 (59.6)	9,097 (28.8)
	Urban	1,972 (8.8)	9 (0.0)	43 (0.2)	653 (2.9)	5,071 (22.5)	14,754 (65.6)	22,502 (71.2)
	**Annual household income**							
	≤60,000 yuan	1,155 (8.6)	6 (0.0)	41 (0.3)	510 (3.8)	3,392 (25.2)	8,344 (62.0)	13,448 (42.6)
	60,000–120,000 yuan	710 (9.3)	4 (0.1)	20 (0.3)	238 (3.1)	1,777 (23.2)	4,907 (64.1)	7,656 (24.2)
	≥120,000 yuan	1,060 (10.1)	4 (0.0)	16 (0.2)	259 (2.5)	2,232 (21.3)	6,924 (66.0)	10,495 (33.2)
Hospital characteristics	**Hospital type**							
	General hospital	1,167 (9.1)	5 (0.0)	35 (0.3)	377 (2.9)	2,683 (20.9)	8,573 (66.8)	12,840 (40.6)
	Traditional Chinese medicine hospital	704 (8.7)	5 (0.1)	22 (0.3)	261 (3.2)	2,210 (27.2)	4,922 (60.6)	8,124 (25.7)
	Maternal and child health	722 (9.9)	3 (0.0)	13 (0.2)	278 (3.8)	1,846 (25.2)	4,451 (60.9)	7,313 (23.1)

	hospital							
	Other specialty hospital	332 (10.0)	1 (0.0)	7 (0.2)	91 (2.7)	662 (19.9)	2,229 (67.1)	3,322 (10.5)
	**Geographic region of hospital**							
	Eastern	1,540 (10.2)	6 (0.0)	29 (0.2)	411 (2.7)	3,391 (22.5)	9,690 (64.3)	15,067 (47.7)
	Central	598 (8.5)	5 (0.1)	23 (0.3)	227 (3.2)	1,413 (20.0)	4,807 (68.0)	7,073 (22.4)
	Western	787 (8.3)	3 (0.0)	25 (0.3)	369 (3.9)	2,597 (27.5)	5,678 (60.0)	9,459 (29.9)
**Health status and clinical characteristics**	**Department visited**							
	Internal medicine	817 (9.0)	5 (0.1)	25 (0.3)	268 (3.0)	2,061 (22.8)	5,860 (64.9)	9,036 (28.6)
	Surgery	448 (10.0)	1 (0.0)	12 (0.3)	131 (2.9)	1,033 (23.1)	2,847 (63.7)	4,472 (14.2)
	Obstetrics and gynecology	613 (9.2)	3 (0.0)	16 (0.2)	230 (3.5)	1,576 (23.8)	4,193 (63.2)	6,631 (21.0)
	Pediatrics	181 (10.1)	0 (0.0)	3 (0.2)	78 (4.4)	474 (26.5)	1,055 (58.9)	1,791 (5.7)
	Otorhinolaryngology	126 (9.8)	1 (0.1)	5 (0.4)	43 (3.3)	311 (24.1)	806 (62.4)	1,292 (4.1)
	Stomatology	100 (4.8)	1 (0.0)	5 (0.2)	41 (2.0)	474 (22.7)	1,467 (70.3)	2,088 (6.6)
	Ophthalmology	135 (8.4)	0 (0.0)	4 (0.2)	67 (4.1)	429 (26.6)	980 (60.7)	1,615 (5.1)
	Oncology	75 (12.3)	0 (0.0)	1 (0.2)	26 (4.3)	128 (20.9)	381 (62.4)	611 (1.9)
	Traditional Chinese medicine	128 (6.3)	0 (0.0)	4 (0.2)	67 (3.3)	491 (24.1)	1,345 (66.1)	2,035 (6.4)
	Other	302 (14.9)	3 (0.1)	2 (0.1)	56 (2.8)	424 (20.9)	1,241 (61.2)	2,028 (6.4)
	**Number of chronic conditions, mean (SD)**	0.4 (0.7)	0.6 (1.2)	0.9 (1.0)	0.5 (0.8)	0.4 (0.7)	0.4 (0.7)	0.4 (0.7)
	**Hypertension**							
	No	2,565 (9.4)	10 (0.0)	66 (0.2)	882 (3.2)	6,401 (23.3)	17,495 (63.8)	27,419 (86.8)
	Yes	360 (8.6)	4 (0.1)	11 (0.3)	125 (3.0)	1,000 (23.9)	2,680 (64.1)	4,180 (13.2)
	**Diabetes**							
	No	2,754 (9.3)	14 (0.0)	65 (0.2)	924 (3.1)	6,922 (23.4)	18,959 (64.0)	29,638 (93.8)
	Yes	171 (8.7)	0 (0.0)	12 (0.6)	83 (4.2)	479 (24.4)	1,216 (62.0)	1,961 (6.2)
	**Cardiocerebrovascular disease**							
	No	2,793 (9.2)	12 (0.0)	65 (0.2)	952 (3.1)	7,106 (23.5)	19,359 (63.9)	30,287 (95.8)
	Yes	132 (10.1)	2 (0.2)	12 (0.9)	55 (4.2)	295 (22.5)	816 (62.2)	1,312 (4.2)

	**Cancer**							
	No	2,843 (9.2)	14 (0.0)	70 (0.2)	960 (3.1)	7,243 (23.6)	19,620 (63.8)	30,750 (97.3)
	Yes	82 (9.7)	0 (0.0)	7 (0.8)	47 (5.5)	158 (18.6)	555 (65.4)	849 (2.7)
	**Chronic respiratory disease**							
	No	2,886 (9.3)	13 (0.0)	70 (0.2)	976 (3.1)	7,291 (23.4)	19,887 (63.9)	31,123 (98.5)
	Yes	39 (8.2)	1 (0.2)	7 (1.5)	31 (6.5)	110 (23.1)	288 (60.5)	476 (1.5)
**Outpatient visit characteristics**	**Type of physician consulted**							
	Renowned specialist	467 (9.4)	2 (0.0)	13 (0.3)	114 (2.3)	971 (19.6)	3,375 (68.3)	4,942 (15.6)
	Chief physician	1,030 (9.1)	5 (0.0)	22 (0.2)	376 (3.3)	2,708 (23.8)	7,235 (63.6)	11,376 (36.0)
	Associate chief physician	516 (8.3)	2 (0.0)	19 (0.3)	195 (3.1)	1,506 (24.3)	3,966 (63.9)	6,204 (19.6)
	Attending physician	524 (8.3)	3 (0.0)	18 (0.3)	186 (3.0)	1,461 (23.2)	4,109 (65.2)	6,301 (19.9)
	Resident physician	70 (10.3)	2 (0.3)	3 (0.4)	34 (5.0)	153 (22.6)	415 (61.3)	677 (2.1)
	Unknown	318 (15.2)	0 (0.0)	2 (0.1)	102 (4.9)	602 (28.7)	1,075 (51.2)	2,099 (6.6)
**Communication and humanistic care experience**	**Satisfied with history taking and disease communication**							
	No	376 (26.2)	4 (0.3)	29 (2.0)	334 (23.3)	464 (32.3)	229 (15.9)	1,436 (4.5)
	Yes	2,549 (8.5)	10 (0.0)	48 (0.2)	673 (2.2)	6,937 (23.0)	19,946 (66.1)	30,163 (95.5)
	**Perceived respect and comfort from healthcare professionals**							
	No	423 (27.1)	6 (0.4)	32 (2.0)	352 (22.5)	526 (33.7)	223 (14.3)	1,562 (4.9)
	Yes	2,502 (8.3)	8 (0.0)	45 (0.1)	655 (2.2)	6,875 (22.9)	19,952 (66.4)	30,037 (95.1)

Table S2. Exploratory interaction analyses for receipt of satisfactory healthy lifestyle guidance

**Interaction term**	**Likelihood ratio χ^2**	**df**	**P for interaction**
Sex × number of chronic conditions	0.041	1	0.839
Age group × satisfaction with history taking and disease communication	3.633	3	0.304

Table S3. Fully adjusted mixed-effects logistic regression analyses of factors associated with receipt of specific healthy lifestyle guidance domains

Category	Characteristic	Dietary guidance		Physical activity guidance		Weight management guidance		Sleep guidance		Smoking cessation and alcohol reduction guidance	
		**OR (95% CI)**	**P value**	**OR (95% CI)**	**P value**	**OR (95% CI)**	**P value**	**OR (95% CI)**	**P value**	**OR (95% CI)**	**P value**
**Patient sociodemographic characteristics**	**Age**										
	≤17 years (Ref.)	Reference		Reference		Reference		Reference		Reference	
	18–35 years	1.101 (0.945, 1.281)	0.216	1.248 (1.083, 1.437)	**0.002**	1.283 (1.102, 1.495)	**0.001**	1.139 (0.962, 1.349)	0.131	3.302 (2.759, 3.951)	**<0.001**
	36–59 years	1.053 (0.909, 1.221)	0.490	1.215 (1.059, 1.394)	**0.005**	1.156 (0.996, 1.342)	0.057	1.146 (0.972, 1.351)	0.104	3.372 (2.829, 4.020)	**<0.001**
	≥60 years	1.070 (0.912, 1.256)	0.406	1.124 (0.969, 1.303)	0.122	1.027 (0.873, 1.207)	0.749	1.132 (0.948, 1.351)	0.171	2.657 (2.208, 3.198)	**<0.001**
**Patient sociodemographic characteristics**	**Sex**										
	Female (Ref.)	Reference		Reference		Reference		Reference		Reference	
	Male	0.941 (0.882, 1.004)	0.066	1.028 (0.969, 1.091)	0.359	0.880 (0.825, 0.938)	**<0.001**	0.784 (0.730, 0.841)	**<0.001**	2.086 (1.960, 2.222)	**<0.001**

**Patient sociodemographic characteristics**	**Senior high school education or above**										
	No (Ref.)	Reference		Reference		Reference		Reference		Reference	
	Yes	1.049 (0.953, 1.154)	0.328	1.146 (1.051, 1.249)	**0.002**	1.080 (0.982, 1.187)	0.112	1.044 (0.942, 1.157)	0.412	1.056 (0.962, 1.159)	0.254
**Patient sociodemographic characteristics**	**Household registration status**										
	Rural (Ref.)	Reference		Reference		Reference		Reference		Reference	
	Urban	1.026 (0.956, 1.102)	0.472	1.030 (0.966, 1.099)	0.362	0.983 (0.917, 1.053)	0.626	0.925 (0.857, 0.998)	**0.043**	0.914 (0.852, 0.980)	**0.012**
**Patient sociodemographic characteristics**	**Annual household income**										
	≤60,000 yuan (Ref.)	Reference		Reference		Reference		Reference		Reference	
	60,000–120,000 yuan	0.971 (0.900, 1.049)	0.458	1.031 (0.962, 1.105)	0.383	1.053 (0.978, 1.134)	0.171	1.081 (0.997, 1.172)	0.058	0.974 (0.903, 1.050)	0.491
	≥120,000 yuan	0.893 (0.829, 0.961)	**0.002**	0.977 (0.914, 1.045)	0.495	1.057 (0.983, 1.136)	0.132	0.981 (0.906, 1.061)	0.629	0.879 (0.816, 0.946)	**<0.001**
**Hospital characteristics**	**Hospital type**										
	General	Refe		Refe		Refe		Refe		Refe	

	hospital (Ref.)	rence		rence		rence		rence		rence	
	Traditional Chinese medicine hospital	1.171 (0.936, 1.465)	0.168	0.903 (0.739, 1.104)	0.320	0.861 (0.692, 1.070)	0.177	1.151 (0.956, 1.386)	0.136	0.929 (0.757, 1.140)	0.481
	Maternal and child health hospital	0.977 (0.772, 1.237)	0.846	0.970 (0.784, 1.200)	0.781	1.670 (1.329, 2.100)	**<0.001**	1.026 (0.842, 1.251)	0.797	0.665 (0.533, 0.829)	**<0.001**
	Other specialty hospital	0.622 (0.454, 0.852)	**0.003**	0.490 (0.367, 0.655)	**<0.001**	0.471 (0.343, 0.647)	**<0.001**	0.680 (0.518, 0.892)	**0.005**	0.801 (0.598, 1.072)	0.135
**Hospital characteristics**	**Geographic region of hospital**										
	Eastern (Ref.)	Reference		Reference		Reference		Reference		Reference	
	Central	0.873 (0.694, 1.099)	0.248	0.937 (0.762, 1.153)	0.539	1.134 (0.906, 1.419)	0.271	1.068 (0.882, 1.294)	0.499	1.165 (0.943, 1.440)	0.156
	Western	0.955 (0.773, 1.181)	0.673	0.919 (0.759, 1.112)	0.386	1.208 (0.982, 1.485)	0.073	1.045 (0.876, 1.248)	0.623	1.130 (0.929, 1.374)	0.221
**Disease burden**	**Number of chronic conditions**										
	Per one-condition increase	0.861 (0.779, 0.952)	**0.003**	1.163 (1.061, 1.274)	**0.001**	1.113 (1.010, 1.227)	**0.030**	1.164 (1.049, 1.292)	**0.004**	1.046 (0.948, 1.154)	0.370
**Disease burden**	**Hypertension**										
	No (Ref.)	Reference		Reference		Reference		Reference		Reference	
	Yes	1.383 (1.2	**<0.001**	0.855 (0.7	**0.015**	0.944 (0.8	0.411	0.851 (0.7	**0.032**	1.159 (1.0	**0.032**

		00, 1.593)		53, 0.970)		24, 1.082)		34, 0.986)		13, 1.325)	
**Disease burden**	**Diabetes**										
	No (Ref.)	Reference		Reference		Reference		Reference		Reference	
	Yes	1.212 (1.030, 1.426)	**0.021**	0.952 (0.823, 1.101)	0.504	1.161 (0.994, 1.355)	0.059	0.883 (0.745, 1.048)	0.155	1.022 (0.876, 1.193)	0.782
**Disease burden**	**Cardiocerebrovascular disease**										
	No (Ref.)	Reference		Reference		Reference		Reference		Reference	
	Yes	1.229 (1.018, 1.484)	**0.032**	0.962 (0.814, 1.137)	0.649	1.000 (0.835, 1.196)	0.997	0.914 (0.752, 1.112)	0.369	1.040 (0.871, 1.241)	0.665
**Disease burden**	**Cancer**										
	No (Ref.)	Reference		Reference		Reference		Reference		Reference	
	Yes	1.495 (1.195, 1.870)	**<0.001**	1.241 (1.025, 1.502)	**0.027**	1.076 (0.877, 1.319)	0.485	1.203 (0.970, 1.491)	0.092	1.132 (0.922, 1.389)	0.235
**Disease burden**	**Chronic respiratory disease**										
	No (Ref.)	Reference		Reference		Reference		Reference		Reference	
	Yes	0.974 (0.757, 1.254)	0.840	0.869 (0.688, 1.097)	0.236	0.829 (0.642, 1.071)	0.151	1.144 (0.881, 1.487)	0.313	1.312 (1.032, 1.668)	**0.027**
**Outpatient visit characteristics**	**Type of physician consulted**										
		Refe		Refe		Refe		Refe		Refe	

	Renowned specialist (Ref.)	rence		rence		rence		rence		rence	
	Chief physician	0.898 (0.819, 0.984)	**0.021**	1.120 (1.033, 1.215)	**0.006**	1.106 (1.013, 1.208)	**0.024**	1.062 (0.964, 1.169)	0.224	1.026 (0.939, 1.121)	0.571
	Associate chief physician	0.773 (0.698, 0.856)	**<0.001**	1.132 (1.034, 1.240)	**0.007**	1.091 (0.989, 1.204)	0.083	1.086 (0.975, 1.209)	0.133	1.043 (0.944, 1.151)	0.407
	Attending physician	0.792 (0.715, 0.877)	**<0.001**	1.001 (0.913, 1.097)	0.990	1.014 (0.918, 1.120)	0.784	1.122 (1.007, 1.251)	**0.037**	0.973 (0.879, 1.075)	0.587
	Resident physician	0.646 (0.525, 0.794)	**<0.001**	0.918 (0.754, 1.118)	0.395	1.271 (1.037, 1.558)	**0.021**	0.989 (0.782, 1.252)	0.929	1.032 (0.832, 1.280)	0.774
	Unknown	0.586 (0.507, 0.676)	**<0.001**	0.861 (0.750, 0.989)	**0.034**	0.925 (0.797, 1.074)	0.307	0.853 (0.721, 1.009)	0.063	0.906 (0.779, 1.053)	0.199
**Outpatient visit characteristics**	**Total cost of this outpatient visit**										
	≤150 yuan (Ref.)	Reference		Reference		Reference		Reference		Reference	
	150–400 yuan	1.090 (1.006, 1.180)	**0.034**	1.036 (0.964, 1.112)	0.336	1.080 (1.002, 1.165)	**0.045**	1.121 (1.031, 1.219)	**0.008**	1.109 (1.025, 1.200)	**0.010**
	>400 yuan	1.036 (0.959, 1.119)	0.367	0.972 (0.906, 1.042)	0.421	0.996 (0.923, 1.073)	0.907	1.048 (0.964, 1.139)	0.274	1.072 (0.992, 1.159)	0.077
**Communication and**	**Satisfied with history**										

**humanistic care experience**	**taking and disease communication**										
	No (Ref.)	Reference		Reference		Reference		Reference		Reference	
	Yes	2.118 (1.838, 2.439)	**<0.001**	1.383 (1.190, 1.606)	**<0.001**	1.311 (1.111, 1.548)	**0.001**	1.525 (1.256, 1.850)	**<0.001**	1.401 (1.184, 1.658)	**<0.001**
**Communication and humanistic care experience**	**Perceived respect and comfort from healthcare professionals**										
	No (Ref.)	Reference		Reference		Reference		Reference		Reference	
	Yes	2.180 (1.901, 2.499)	**<0.001**	1.812 (1.563, 2.100)	**<0.001**	1.851 (1.567, 2.185)	**<0.001**	1.533 (1.274, 1.845)	**<0.001**	1.415 (1.204, 1.664)	**<0.001**

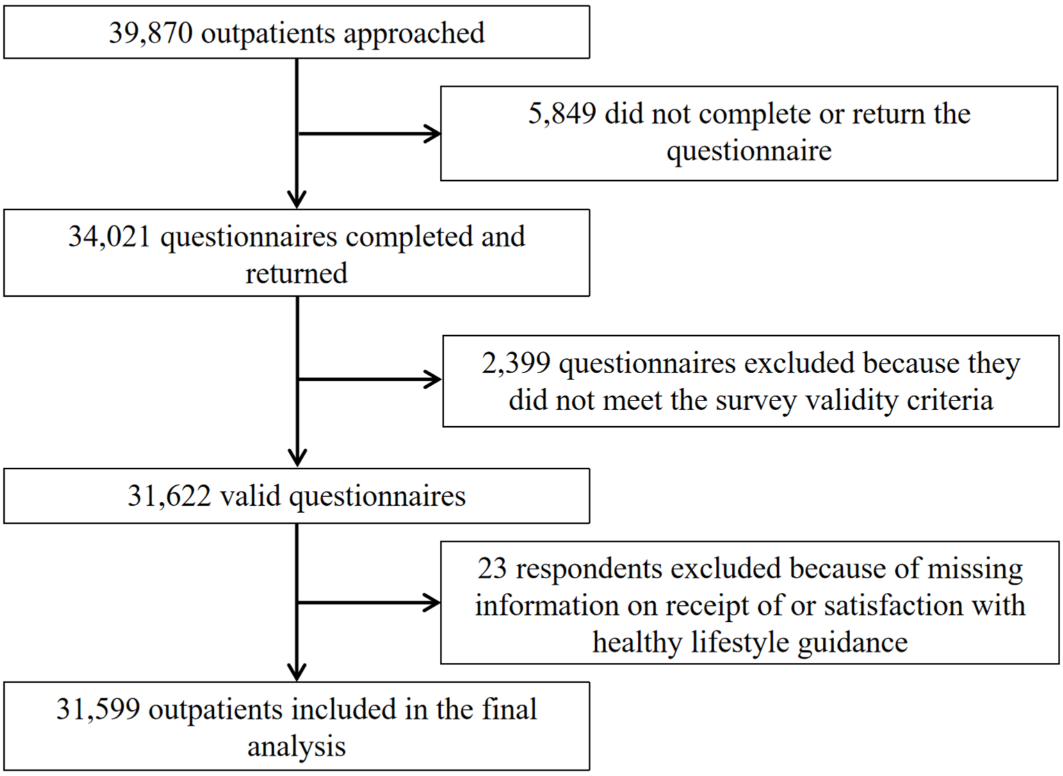

Figure S1. Flow diagram of outpatient recruitment and derivation of the final analytical sample.
